# Type 2 Inflammation in Eosinophilic Esophagitis: From Pathophysiology to Therapeutic Targets

**DOI:** 10.3389/fphys.2021.815842

**Published:** 2022-01-12

**Authors:** Francesca Racca, Gaia Pellegatta, Giuseppe Cataldo, Edoardo Vespa, Elisa Carlani, Corrado Pelaia, Giovanni Paoletti, Maria Rita Messina, Emanuele Nappi, Giorgio Walter Canonica, Alessandro Repici, Enrico Heffler

**Affiliations:** ^1^Personalized Medicine, Asthma and Allergy, IRCCS Humanitas Research Hospital, Rozzano, Italy; ^2^Department of Biomedical Sciences, Humanitas University, Pieve Emanuele, Italy; ^3^Digestive Endoscopy Unit, Department of Gastroenterology, IRCCS Humanitas Research Hospital, Rozzano, Italy; ^4^Department of Medical and Surgical Sciences, University “Magna Graecia” of Catanzaro, Catanzaro, Italy

**Keywords:** eosinophilic esophagitis, type 2 inflammation, therapeutic targets, precision medicine, pathophysiology

## Abstract

Eosinophilic esophagitis (EoE) is a chronic immune-mediated disease of the esophagus characterized clinically by symptoms related to esophageal dysfunction and histologically by eosinophil-predominant inflammation, whose incidence is rising. It significantly affects patients’ quality of life and, if left untreated, results in fibrotic complications. Although broad consensus has been achieved on first-line therapy, a subset of patients remains non-responder to standard therapy. The pathogenesis of EoE is multifactorial and results from the complex, still mostly undefined, interaction between genetics and intrinsic factors, environment, and antigenic stimuli. A deep understanding of the pathophysiology of this disease is pivotal for the development of new therapies. This review provides a comprehensive description of the pathophysiology of EoE, starting from major pathogenic mechanisms (genetics, type 2 inflammation, epithelial barrier dysfunction, gastroesophageal reflux, allergens, infections and microbiota) and subsequently focusing on the single protagonists of type 2 inflammation (involved cells, cytokines, soluble effectors, surface proteins and transcription factors) that could represent present and future therapeutic targets, while summarizing previous therapeutic approaches in literature.

## Introduction

Eosinophilic esophagitis (EoE) is a chronic immune-mediated disease of the esophagus characterized clinically by symptoms related to esophageal dysfunction and histologically by eosinophil-predominant inflammation (Dellon and Hirano, 2018).

Eosinophilic esophagitis can affect all age groups, with an incidence peak between the third and the fifth decade of life. Its estimated prevalence is 30–100/100.000 in the adult population and 29–42/100.000 in the pediatric population (Soon et al., 2013; Dellon et al., 2014a; Arias et al., 2016). EoE is most frequent in males, with a male-to-female ratio of about 3:1 (Hruz, 2014) and a predilection for Caucasian ethnicity (Arias et al., 2016).

Since its relatively new recognition in the ‘70s, EoE incidence has risen rapidly over the last 15 years both in the US (Prasad et al., 2009) and in Europe (Hruz et al., 2011; van Rhijn et al., 2013) and EoE is now recognized as the first cause of dysphagia in the adult population (Moawad et al., 2014). The reasons for this recent increase are still debated and, while increased recognition and raised awareness undoubtedly contributed, data are consistent with a true increase (Dellon, 2014; Dellon et al., 2015).

Up to 80% of patients have a personal history of atopic comorbidities, such as allergic rhinitis, asthma, food allergy and atopic dermatitis (AD) (Franciosi et al., 2009). Other associated diseases comprehend celiac disease, inherited connective tissue disorders (CTDs), type 1 diabetes, cystic fibrosis, autism, esophageal atresia and monogenic diseases (i.e., autosomal dominant hyper-IgE syndrome, Netherton syndrome, Severe atopic syndrome associated with metabolic wasting -SAM) (Abonia et al., 2013; Guarino et al., 2016; Votto et al., 2020).

The main clinical manifestations of EoE in adults are dysphagia and food impaction after ingestion of solid foods. Other less common symptoms are chest pain and refractory heartburn and regurgitation. In children and infants, symptoms might be more subtle with failure to thrive, vomiting, nausea, regurgitation, abdominal pain, food aversion, and feeding problems (Noel et al., 2004; Mukkada et al., 2010).

**FIGURE 1:**
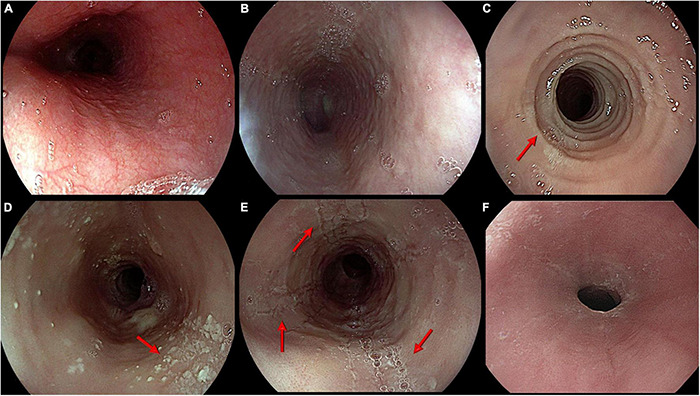
Main endoscopic features of eosinophilic esophagitis. From top left: **(A)** Normal appearance of esophageal mucosa; **(B)** Edema. Pale mucosa with attenuation of the normal vascular pattern; **(C)** Rings. Trachealized esophagus with multiple concentric rings (arrow); **(D)** Exudates. Whitish small plaques not washable through water jet (arrow); **(E)** Furrows. Typical longitudinal furrows (arrows); **(F)** Stricture. Narrowing of esophageal lumen not passable by a standard scope (diameter around 9 mm).

Compensation mechanisms, such as prolonged mastication and assumption of liquids during meals, frequently lead to diagnostic delay (Reed et al., 2018; Lenti et al., 2021). The endoscopic appearance of EoE can show mucosal edema, mucosal rings (trachealization), exudates, linear furrows, and strictures (Sherrill and Rothenberg, 2014). However, up to one-third of EoE patients have a macroscopically normal endoscopy (Liacouras et al., 2011; Figure 1).

The current consensus criteria for EoE diagnosis include signs and symptoms of esophageal dysfunction and a eosinophil-predominant inflammation of the esophagus confirmed histologically by a peak count equal or higher than 15 eosinophils per high-power field, in the absence of other causes of esophageal eosinophilia (including eosinophilic gastritis -EG and enteritis – EGE, Leśniowski-Crohn disease, parasitic infection, achalasia, hypereosinophilic syndrome, hypersensitivity to medicines, CTD, vasculitis, graft-versus-host disease, pemphigus) (Lucendo et al., 2017). Natural history studies revealed that EoE is a chronic disease that significantly impacts on quality of life, including vitality and general health scores (van Rhijn et al., 2014a) and, if left untreated, results in continued inflammation (Dellon and Hirano, 2018) and complications such as strictures may develop (Schoepfer et al., 2013; Dellon et al., 2014b).

**FIGURE 2:**
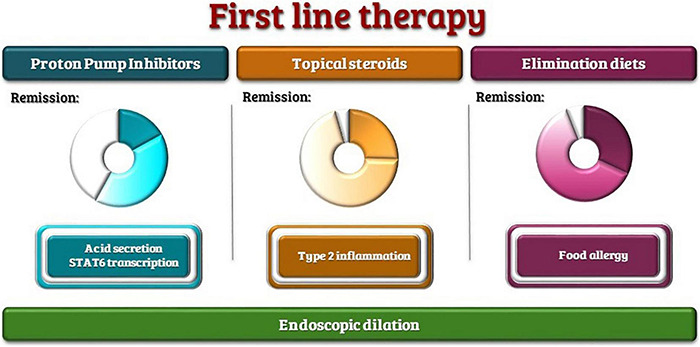
First-line therapies with the range of reported remission rates and therapeutic targets.

Although broad consensus has been achieved on first-line therapies (Dellon and Hirano, 2018), that involve proton pump inhibitors (PPIs), swallowed topical steroids and elimination diets (Figure 2), a subset of patients remains non-responder to standard therapy.

A deep understanding of the pathophysiology of this disease is pivotal for the development of new therapies.

The aim of this review is to provide a comprehensive description of the pathophysiology of EoE with a particular focus on therapeutic targets and results from previous therapeutic approaches in literature.

## Major Pathogenic Mechanisms

**FIGURE 3:**
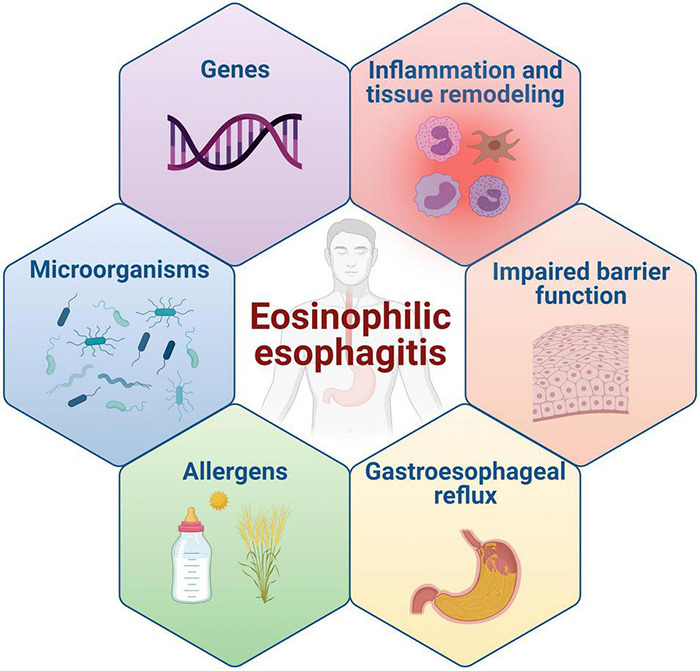
Main pathogenetic mechanisms of EoE.

The pathogenesis of EoE is multifactorial and results from the complex, still mostly undefined, interaction between genetics and intrinsic factors, environment, and antigenic stimuli (Lim et al., 2011; Kottyan et al., 2014; Figure 3).

### Genetics

Eosinophilic esophagitis occurs in family members in a non-Mendelian pattern, indicating a complex heritability (Straumann et al., 2016).

Male predominance, family history, twin concordance and genomewide association studies (GWAS) suggest that genetics contribute to the development of EoE (Alexander et al., 2014). Familial studies have identified a 44% concordance between monozygotic twins and 30% concordance in dizygotic twins, with familiarity proven in 7–10% of patients and a sibling recurrence ratio of 80/10.000 (Sherrill and Rothenberg, 2011; Guarino et al., 2016). The heritability risk is estimated to be 2% with a reported relative risk ratio for EoE in family members of 10–64, and is greater for male relatives (Alexander et al., 2014).

Candidate gene studies, GWAS and phenome-wide association studies (PheWas) identified multiple genes possibly contributing to the development; these include genes involved in the so-called “Type 2 (T2) inflammation” pathways, epidermal differentiation and barrier function. Interestingly, also some defects in mitochondrial function genes (dehydrogenase E1, and Dehydrogenase E1 and Transketolase Domain Containing 1 – DHTKD1) were associated with EoE (Henderson et al., 2014).

It should also be pointed out that many of these genetic loci were identified in other atopic diseases, including AD (c11orf30, filaggrin – FLG, desmoglein-1 – DSG-1, and serine peptidase inhibitor Kazal type 5 – SPINK5), asthma (Thymic stromal lymphopoietin – TSLP, c11orf30, C-C Motif Chemokine Ligand 26 – CCL26), allergic sensitization (TSLP, c11orf30, Signal Transducer and Activator Of Transcription 6 – STAT6) and allergic rhinitis (TSLP, c11orf30) (Doucet-Ladevèze et al., 2019; Lyles and Rothenberg, 2019).

### Type 2 Inflammation

**FIGURE 4:**
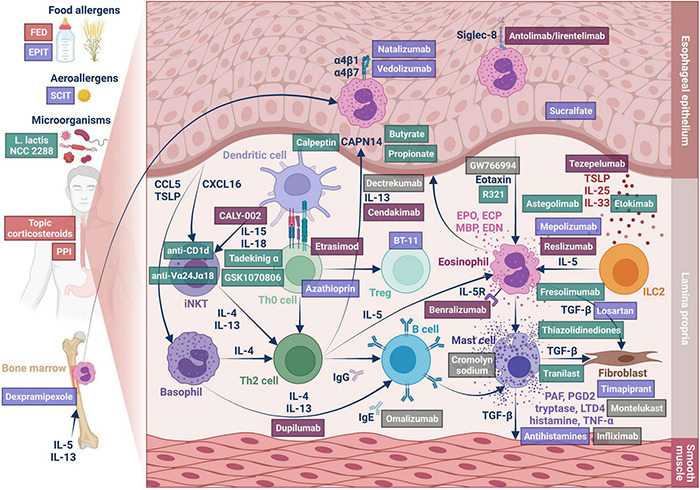
Type 2 inflammation in EoE. The figure represents the involved cells and mediators and the therapeutic approaches described in the review. The drugs are colored as follows: Red tag: first-line therapy, Plum tag: orphan drugs, Lilac tag: drugs with reported results on human patients, Green tag: drugs without reported results on human patients, Gray tag: drugs that failed to obtain significant results on human patients.

Eosinophilic esophagitis inflammation presents all the hallmarks of a type 2 immunological response (Figure 4).

Following a still debated likely initial stimulus, epithelial and dendritic cells start to express epithelial-derived cytokines (the so-called “alarmins,” namely interleukin 25 – IL25, interleukin 33 – IL33, and TSLP), leading to homing and retention of immune cells, such as invariant natural killer T (iNKT) cells, adaptive CD4^+^ effector memory T-helper 2 (Th2) cells and innate lymphoid type 2 cells (ILC2) (Lexmond et al., 2014; Shukla et al., 2015).

Their activation, in turn, leads to the secretion of key cytokines, such as interleukins 13 (IL-13), 4 (IL-4), and 5 (IL-5), that ultimately lead to the migration of eosinophils to the esophagus and their degranulation with the release of many molecules responsible of tissue remodeling, as well as damage of the epithelial barrier.

#### Targeting Type 2 Inflammation as a Whole: Corticosteroids

**Steroids** represented the first treatment approach for EoE (Liacouras et al., 1998). Initially, systemic steroids were employed; however, topical steroids were subsequently proven equally effective, while sparing systemic adverse events (Faubion et al., 1998; Schaefer et al., 2008). Thus, systemic steroids are currently reserved for critical patients with severe dysphagia or significant weight loss (Gonzalez-Cervera and Lucendo, 2016; Lucendo et al., 2017). Swallowed topical corticosteroids, mainly fluticasone propionate and budesonide, currently represent one of the most effective first-line therapies for EoE in both children and adults (Navarro et al., 2021; Rokkas et al., 2021).

Glucocorticoid treatment reduces interleukin-13 transcription and the EoE transcriptome *in vivo* (Blanchard et al., 2007), reduces or even abolishes the eosinophilic infiltrate in 39–100% of patients (Hao et al., 2021), reduces T cell infiltration (Teitelbaum et al., 2002), downregulates mast cell associated genes (Hsu Blatman et al., 2011) and even decreases fibrosis (Aceves et al., 2010b) and restores esophageal motility (Nennstiel et al., 2016; Nakajima et al., 2017).

Swallowed topical steroids seem to be safe; the few adverse effects are superficial esophageal candidiasis (described in up to 10% of patients; it responds to specific treatment) and rarely adrenal axis suppression, bone demineralization, and diminished growth (Bagiella and Chehade, 2016).

Current research is focused on the development of formulations that enable optimal esophageal delivery (Racca et al., 2021).

### Epithelial Barrier Dysfunction

Human esophageal mucosa consists of a multilayer squamous non-keratinized epithelium, which, together with the mucus layer, protects tissues from microorganisms and mechanical and chemical insults (Squier and Kremer, 2001).

Various assessments on esophageal tissues from patients with EoE have demonstrated the presence of an altered epithelial barrier function (van Rhijn et al., 2014c), with reduced transepithelial resistance and impedance (Katzka et al., 2015; Patel and Vaezi, 2017; Warners et al., 2017a,b). Histological characteristics comprehend dilated interepithelial spaces, basal cell hyperplasia (Mueller et al., 2006; Ravelli et al., 2006; Katzka et al., 2014), decreased desmosomes (Capocelli et al., 2015) and a profound loss of esophageal tissue differentiation (Rochman et al., 2017).

The principal factors involved in these alterations are the epidermal differentiation complex (EDC), FLG and Calpain (CAPN)14.

The EDC, on the 1q21 locus, is a cluster of genes involved in epithelial differentiation (South et al., 1999) and displays the most abundant dysregulation in the EoE transcriptome (Blanchard et al., 2010). EDC genes include FLG, involucrin, and many small proline-rich repeat (SPRR) family members (Blanchard et al., 2010).

Filaggrin encodes an intracellular protein involved in the aggregation of keratin filaments and interestingly, its dysfunction has been associated with AD (Fallon et al., 2009; O’Regan et al., 2009), a disease that frequently co-occurs with EoE.

Other downregulated junctional proteins in the esophageal mucosa of patients with EoE include *E*-cadherin, claudin-1 and 7 (Abdulnour-Nakhoul et al., 2013; Nguyen et al., 2018), zonulin-1 (Katzka et al., 2014), and desmoglein-1 (DSG1) (Sherrill et al., 2014a).

CAPN14 is an intracellular regulatory protease, member of the classical calpain subfamily, involved in the integrity of the esophageal epithelial barrier. This protease is exclusively expressed in the esophageal mucosa (Sleiman et al., 2014; Kottyan et al., 2021) and is dynamically regulated by IL-13 (Kottyan et al., 2014). Higher levels of CAPN14 expression, such as seen in active EoE (Sleiman et al., 2014; Kottyan et al., 2021), are associated with the downregulation of DSG1, FLG, and zonulin, all involved in epithelial barrier function (Sleiman et al., 2014). Proteases also seem to play a role in EoE. Serine protease inhibitors (SERPINs), serine protease inhibitors, Kazal type (SPINKs) and Kallikrein serine peptidases (LRRC31) have been shown to be dysregulated in EoE (D’Mello et al., 2016; Rochman et al., 2017; Azouz et al., 2018, 2020), potentially contributing to epithelial damage.

It is still debated whether these alterations might be a cause or a consequence of EoE. Usually, food allergens are large molecules, with a molecular weight between 3 and 90 kDa, and are unable to cross the healthy esophageal epithelium. It has been postulated that altered epithelial permeability could lead to a permissive environment that facilitates contact between antigens and the immune system leading to the development of inflammation, but it can also be considered a consequence of esophageal inflammation.

On one side, eosinophil products have a known cytotoxic effect. Moreover, IL-13 and transforming growth factor β1 (TGF-β1), key cytokines in EoE, impact epithelial differentiation and cellular junction formation, downregulating EDC expression (Blanchard et al., 2010). Of note, treatment with FEDs or steroids has shown to reverse FLG downregulation (Politi et al., 2016).

Moreover, Major Basic Protein (MBP) and TGF, secreted by eosinophils (Gleich et al., 1979), promote extracellular matrix damage by increasing mast cells’ secretion of proteolytic enzymes, such as tryptase and chymase.

However, about 2% of EoE transcriptional alterations do not resolve upon disease remission (Blanchard et al., 2007) with persistence of dysregulations of cadherin-like 26 (CDH26), uroplakin 1B (UPK1B), periostin (POSTN), and DSG1, all involved in epithelial homeostasis (Blanchard et al., 2007).

Moreover, multiple genetic variants in epithelial barrier-related genes have been associated with an increased risk of developing EoE in life. These include two coding variants in FLG gene (R501X and 2282del4), associated with EoE irrespective of atopic status (Blanchard et al., 2010), a genetic risk locus located on chr19q13.11 that encodes several genes (ANKRD27, PDCD5) involved in apical transport in esophageal epithelial cells, wound healing (Tamura et al., 2011; Fukuda, 2016), transcriptional regulation, DNA damage response, and cell cycle control (Li et al., 2016), a variant in the 10p14 locus in an intron of Inter-α-trypsin inhibitor heavy chain 5 (ITIH5), a gene that encodes for a serine protease inhibitor and has been also linked to eosinophilic granulomatosis with polyangiitis (Kottyan et al., 2021), and a locus on chr2p23.1, involving the CAPN14 gene, was found to be highly associated with EoE in multiple studies (Kottyan et al., 2014, 2021; Sleiman et al., 2014).

Moreover, a Mendelian association was found between EoE and SAM syndrome, caused by homozygous mutations in DSG1 (Ryu et al., 2020), and Netherton syndrome, caused by autosomal dominant loss-of-function mutations in the protease inhibitor SPINK5 leading to unrestricted protease activity of KLK5 and KLK7 (Paluel-Marmont et al., 2017). Indeed, a murine model of EoE induced by epicutaneous ovalbumin (OVA) sensitization has pointed out that topical allergen application on unstripped skin induced experimental EoE in FLG-deficient mice but not in wild-type control mice (Venturelli et al., 2016).

#### Epithelial Barrier as a Potential Direct Therapeutic Target

There is scarce literature regarding epithelial barrier-targeted treatments in EoE.

**Sucralfate**, the aluminum salt of sucrose sulfate, is a known “cytoprotective” agent whose mechanism of action supposedly consists in the protection of eroded areas, induction of prostaglandin production and increase of growth factors bioavailability, leading to improved vascular flow and mucus production (Masuelli et al., 2010). This compound has also been shown to reduce the permeability of esophageal mucosa to hydrogen ions (Orlando and Powel, 1984), which elicited its proposal as a potential therapy for EoE. An early phase 1 clinical trial (NCT02353078) on the effect of sucralfate is listed as completed in 2016 on Clinicaltrials.gov^1^ but no result or publication is available so far.

**Butyrate and propionate**, short chain fatty acids (SCFAs) produced by microbial macronutrients fermentation, able to modulate host immune responses and human dendritic cell (DC) function, recently demonstrated *in vitro* capacity of reversing IL-13-driven impairment of esophageal epithelial barrier upregulating FLG and DSG1 expression and reducing CCL26 and CAPN14 expression (Kleuskens et al., 2021). However, no *in vivo* study is available.

Finally, in a recent study of an asthma mouse model, inhibition of CAPN by **calpeptin** was able to strongly reduce bronchial reactivity, bronchoalveolar lavage (BAL) fluid eosinophilia, subepithelial fibrosis and the expression of IL-4, IL-5, IL-13, TGF-β1, and ova-specific immunoglobulin E (Aich et al., 2012), suggesting its therapeutic potential in EoE.

### The Role of Gastroesophageal Reflux

The role of gastroesophageal reflux disease (GERD) in the pathogenesis of EoE is to date controversial. It is now universally accepted that the two diagnoses are not mutually exclusive and, despite their coexistence might be unrelated and due to the high prevalence of GERD in the general population, it has been suggested that the two diseases might have a more complex relationship (Spechler et al., 2007).

On one side, GERD can increase the permeability of the esophageal epithelium to food allergens (Votto et al., 2020) and promote inflammation and eosinophil recruitment (Tobey et al., 2004; Untersmayr and Jensen-Jarolim, 2006). On the other hand, the products of eosinophilic inflammation can reduce esophageal clearance by affecting smooth muscle contraction and esophageal compliance.

It is long known that GERD, both basic and acid, can stimulate squamous epithelial cells to produce eosinophil chemoattractants, such as IL-8 and RANTES (Regulated upon Activation Normal T cell Expressed and Secreted), leading to a mild esophageal eosinophilia (Winter et al., 1982; Isomoto et al., 2003). *In vitro* acid exposure upregulates eotaxins 1, 2, and 3 and macrophage inflammatory protein 1a (MIP-1a) (Ma et al., 2012) by esophageal squamous epithelial cells, promoting eosinophil recruitment (Luster and Rothenberg, 1997).

Eosinophils, on the other hand, produce vasoactive intestinal peptide (VIP) and IL-6 which reduce lower esophageal sphincter (LES) tone and weaken esophageal muscle contraction predisposing to reflux (Cao et al., 2004, 2006; Sarbinowska and Wasko-Czopnik, 2020). About 25–76% of EoE patients present esophageal motility abnormalities (van Rhijn et al., 2014b; Nennstiel et al., 2016; Spechler et al., 2018), mainly hypo-contractile abnormalities such as ineffective esophageal motility, absent contractility, hypotensive LES (van Rhijn et al., 2014b).

Post reflux swallow-induced peristaltic wave (PSPW), an index of esophageal chemical clearance, is reduced in EoE patients. Proton pump inhibitors (PPI) treatment improved all reflux parameters in PPI-responsive patients, including PSPW. In contrast, in PPI-unresponsive patients, PPI treatment improved all reflux parameters except for PSPW (Frazzoni et al., 2019).

Moreover, eosinophils secrete profibrotic products (such as MBP, TGF-β, IL-13, VEGF, and IL-8) that can induce tissue remodeling, interfering with lower esophageal sphincter function and peristalsis (Cheng et al., 2012).

Gastroesophageal reflux disease patients also display esophageal barrier alterations similar to EoE patients, such as dilated intercellular spaces (DISs), basal cell hyperplasia (Zentilin et al., 2005), and tight junction proteins alterations (e.g., claudin-1) (Vieth et al., 2016).

#### Gastroesophageal Reflux as a Therapeutic Target

**PPIs** are an established first-line therapy for EoE, with a reported efficacy in 50% of pediatric and adult patients (Lucendo et al., 2016). However, beyond their blocking effect of gastric acid secretion, PPIs have demonstrated an anti-inflammatory effect on STAT6 (Molina-Infante et al., 2014) and will be therefore treated in the correspondent paragraph.

No other anti-reflux therapy has been tested on EoE to our knowledge.

### Tissue Damage and Remodeling

Natural history studies revealed that, in line with other eosinophilic inflammatory conditions, patients whose inflammation is not controlled risk a gradual shift toward fibrosis (Schoepfer et al., 2013; Dellon, 2014).

Fibrotic remodeling occurs in both pediatric (Aceves et al., 2007; Chehade et al., 2007) and adult (Straumann et al., 2010a; Lucendo et al., 2011a) EoE patients. The persistence of activated fibroblasts was reported even after disease remission (Nhu et al., 2020).

IL-13, IL-4, IL-5, eotaxin-3, periostin, and TGFB1, together with eosinophils products like MBP, all have a role in tissue remodeling (Li-Kim-Moy et al., 2011; Schoepfer et al., 2013; Dellon et al., 2014b).

FGF9, CCL18, and Matrix metalloproteases (MMPs) are other important factors in fibroblast activation and extracellular matrix deposition (Lucendo et al., 2011a; Beppu et al., 2015).

Epithelial-mesenchymal transition (EMT), a process in which epithelial cells lose typical epithelial characteristics acquiring myofibroblasts’ functions, was suggested to play a major role in fibrosis (Kalluri and Weinberg, 2009). This process increases the number of activated fibroblasts and myofibroblasts involved in the extracellular matrix production (Kalluri and Weinberg, 2009; Roman et al., 2010; Kagalwalla et al., 2012; Muir et al., 2013) and can be induced by TGF-β, MMP-9, IL-13, and MBP released by eosinophils or damaged epithelium (Zeisberg, 2008; Kagalwalla et al., 2012).

Therapeutic approaches aimed at remodeling are targeted to the individual effectors and will be treated in individual paragraphs.

### Allergens

Several lines of evidence support the theory of EoE as an allergen-driven disease.

#### Food Allergens

The concept of EoE as a food antigen-triggered disorder was initially based on the finding that, in children, feeding with elemental formula caused histologic remission (Kelly et al., 1995). This conception was then confirmed by the efficacy of FEDs, together with reports of onset of EoE in 2.7% of patients undergoing oral immunotherapy for food allergy desensitization (peanut, milk, egg) with subsequent resolution after suspension (Lucendo et al., 2014; Nilsson et al., 2021; Wright et al., 2021). Some other less repeated but interesting studies reported the presence of gluten deposits in esophageal surface of adult patients (Marietta et al., 2017), the presence of T killer cells able to recognize lipids and produce IL-4 and IL-13 after stimulation with cow milk lipids in patients with active EoE (Jyonouchi et al., 2013) and Th2 cells with CD154 + IL5 + phenotype in the peripheral blood of active EoE patients (but not in control atopic patients or inactive EoE patients) that can be stimulated by milk antigens in milk-induced EoE patients (Cianferoni et al., 2018).

Lately, a novel syndrome, referred to as “food-induced immediate response of the esophagus” (FIRE), consisting in an unpleasant or painful sensation, unrelated to dysphagia, occurring immediately after esophageal contact with specific foods, was observed in EoE patients and theorized to derive from a different, still undefined, physiopathological mechanism (Biedermann et al., 2021).

##### Food Allergy as a Therapeutic Target

Food elimination diet (FED) therapy is today a recognized first-line therapy for EoE.

This approach’s efficacy depends on the extension of eliminated food antigens.

The elemental diet is now known to be efficacious in both children (Markowitz et al., 2003) and adults (Peterson et al., 2013; Warners et al., 2017c), leading to remission in around 70–90% of patients (Arias et al., 2014) and outperforms all other dietary-based strategies. However, its use in clinical practice is inhibited by the poor palatability (Liacouras et al., 2005), psycho-social impact (Franciosi et al., 2012; Slae et al., 2015), cost and developmental impact (Delaney and Arvedson, 2008) and is now used only in refractory patients or as a short-term approach to rapidly induce remission, especially in children (Arias and Lucendo, 2014; Mukkada and Furuta, 2014).

The empiric elimination of the six most common food allergens (cow’s milk proteins, wheat, egg, soy, peanut, fish/seafood) has a reported efficacy in around 70% of pediatric patients and 50% of adult patients (Kagalwalla et al., 2006; Arias et al., 2014; Philpott et al., 2016) but should be followed by sequential reintroduction of foods with repeated endoscopy and biopsies to identify the specific food or foods triggering EoE in each patient. Some reduced empiric diets eliminating 4 (milk, wheat, egg, and soy) (Kagalwalla et al., 2017), two foods (milk and gluten) or one food with a step-up approach in non-responders have been proposed (Molina-Infante et al., 2018a). FEDs based on skin prick test and atopy patch test were reported to lead to remission a high portion of patients in some studies (Spergel et al., 2012a; Terrados et al., 2020), but a sequent meta-analysis showed an overall efficacy of 45.5% and are not currently recommended, together with approaches based on basophil activation tests, serum food-specific IgG, component-resolved diagnosis and esophageal prick tests (van Rhijn et al., 2015; Philpott et al., 2016; Warners et al., 2018).

Recently, a combination of a peripheral blood CD4 + T-cell proliferation assay and food-specific tissue IgG4 levels was reported reduce median peak eosinophil counts but a low histologic remission rate (21%) (Dellon et al., 2019b).

Epicutaneous immunotherapy (EPIT) is an emerging treatment for food allergy (Bégin et al., 2021). A pilot study on pediatric patients with milk-induced EoE treated with EPIT found no significant difference between groups for the maximum eosinophil count at the end of the study. However, when only the subset of patients without major protocol deviations was selected, a significant reduction in eosinophil count was reported, with a 47% remission rate (<15 eosinophils/HPF) and a 36% deep remission rate (<5 eosinophils/HPF) (Spergel et al., 2020), that persisted after 2 years (Spergel et al., 2021).

#### Aeroallergens

The association of EoE with allergic rhinoconjunctivitis and asthma and especially the high prevalence of concurrent sensitization to aeroallergens (Capucilli et al., 2018), together with repeated reports of seasonal exacerbations of the disease (Fogg et al., 2003; Almansa et al., 2009; Ram et al., 2015; Reed et al., 2019), have raised the idea that aeroallergens could have a causative role in EoE.

Indeed, murine models using *Aspergillus fumigatus*, cockroach, or dust mite antigen demonstrate that aeroallergens can cause esophageal eosinophilia (Mishra et al., 2001; Rayapudi et al., 2010). Moreover, aeroallergens can induce EoE in humans when administered in the form of sublingual immunotherapy (Miehlke et al., 2013; Rokosz et al., 2017; Fujiwara et al., 2021) and house mites’ antigens have been found in the esophageal mucosa of EoE patients (Ravi et al., 2019), but the topic has not been further investigated.

##### Hypersensitivity to Aeroallergens as a Therapeutic Target

Some case reports and small series report the efficacy of subcutaneous immunotherapy for aeroallergens in EoE resolution (De Swert et al., 2013; Iglesia et al., 2021) but no clinical trial has been reported.

### Infections and Microbiota

The finding that premature delivery, cesarean birth, early antibiotic exposure, lack of breastfeeding and lack of early microbial exposure are risk factors for the development of EoE (Radano et al., 2014; Jensen and Dellon, 2018) has revived the idea of the Hygiene Hypothesis also in EoE (Mennini et al., 2021).

Indeed, Helicobacter pylori infection, known to increase Th1 and Th17 response, thus reducing Th2-skewing (Shi et al., 2010), has been linked to a reduction in the chance of developing EoE in many, mostly retrospective, studies (Furuta et al., 2013; Shah et al., 2019). However, the data has not been confirmed by a prospective study involving a large number of children (Molina-Infante et al., 2018b).

On the contrary, herpes simplex virus infection might trigger EoE in immunocompetent adults and children (Squires et al., 2009; Žaja Franulović et al., 2013; Fritz et al., 2018).

The oral and esophageal microbiota in patients with EoE has also been studied and results report a shift from a normally predominantly Gram-positive population (*Streptococcus* spp. and *Atopobium* spp.) to an increase in Gram-negative *Haemophilus* spp. and *Proteobacteria* (*Neisseria* spp. and *Corynebacterium* spp.) (Yang et al., 2009; Benitez et al., 2015; Hiremath et al., 2019). However, the difference was not significant between active and non-active EoE in most studies, and it is still unknown if the alteration could have a causative role or represent just a consequence of the altered microenvironment in this disease.

#### Infections and Microbiota as Therapeutic Targets

A single study on a murine model elicited by epicutaneous sensitization with *Aspergillus fumigatus* protein extract, reported a significant decrease of esophageal eosinophilia after supplementation with *Lactococcus lactis NCC 2287*, a strong inducer of the immunomodulatory cytokine IL-10 and an inhibitor of the eosinophil survival cytokine IL-5 (Holvoet et al., 2016). However, this effect was noticeable only when *L. lactis* was used after induction of the inflammation, and not when administered as a preventive strategy. No further studies were found on the subject.

## Focus on Type 2 Inflammation: The Protagonists

### Involved Cells

#### Eosinophils

Eosinophils are the hallmark of EoE.

They present receptors for eotaxin-1, -2, -3, RANTES, MIP-1α, MCP-2, -3, -4, and lipid mediators like Platelet Activating Factor (PAF), Leukotriene B4 (LTB4) and C4 (LTC4) (Shukla et al., 2015) and, upon activation, release cationic granule proteins, reactive oxygen species, lipid mediators, enzymes, growth factors and cytokines, largely responsible for the esophageal modifications seen in EoE.

Eosinophilic peroxidase (EPO), eosinophil cationic protein (ECP), and MBP have cytotoxic effects on the epithelium (Talley et al., 1992) and, together with EPO products (hydrogen peroxide and halide acids) and superoxide generated by the respiratory burst oxidase enzyme pathway (Talley et al., 1992; Reed et al., 2019), contribute to mucosal barrier impairment.

Eosinophils can alter esophageal motility through multiple mechanisms: MBP directly causes vagal muscarinic M2 receptor dysfunction, thus increasing smooth muscle reactivity (Lewis et al., 1990), and, together with TGF-β1, can lead to smooth muscle hyperplasia and hypercontractility; leukotriene D4, prostaglandin F2 alpha, and thromboxane B2 can cause contraction of esophageal muscle (Daniel et al., 1979; Kim et al., 1998; Rosenberg et al., 2013), while IL-6 and IL-13 reduce its amplitude (Rieder et al., 2014). Moreover, eosinophil-derived neurotoxin (EDN) and ECP have ribonuclease activity, toxic for neurons (Gleich et al., 1993).

MBP, TGF-β, IL-13, VEGF and IL-8 are also major factors contributing to EMT, remodeling and fibrosis (Zeisberg, 2008; Cheng et al., 2012; Kagalwalla et al., 2012).

Eosinophils stimulate inflammatory cell recruitment and activation of mast cells, basophils, T cells, fibroblasts, all involved in EoE pathophysiology, and can even act as antigen-presenting cells (Gleich et al., 1993; Mulder et al., 2008; Davis and Rothenberg, 2013).

They can produce IL-1, IL-3, IL-4, IL-5, IL-13; GM-CSF; TGF-β; tumor necrosis factor-α (TNF-α); RANTES (regulated on activation, normal T cells expressed and secreted); macrophage inflammatory protein 1 (MIP-1); and eotaxin (Klion et al., 2020), whose activity will be treated separately.

##### Eosinophils as a Therapeutic Target

**Benralizumab** is a fully humanized afucosylated anti-human IL5-Rα antibody approved for severe asthma (Bleecker et al., 2016; FitzGerald et al., 2016). Its mechanism of action consists in recognition of the IL-5Rα subunit of the IL-5 receptor and activation of FcγRIIIa receptor on natural killer cells, thus rapidly depleting eosinophils (Kolbeck et al., 2010; Laviolette et al., 2013). A randomized, double-blind, placebo-controlled clinical trial (NCT03473977) to evaluate the efficacy of benralizumab in adults and adolescents with EG has recently been concluded,^2^ but the results have not yet been published. Results available in Clinicaltrials.gov report a significantly higher rate of histological remission (77%) vs. placebo (8%), and an improvement in the histologic score with non-significant improvement in endoscopic score and symptoms, in the absence of serious adverse events (SAE), while no statistical analysis is available on non-serious adverse events (AEs).

An ongoing phase 3 trial (NCT04543409) is currently assessing its efficacy in adult and adolescent EoE patients.^3^

Of note, in August 2019, the US Food and Drug Administration (FDA) has granted Orphan Drug Designation to benralizumab for the treatment of EoE.^4^

**Dexpramipexole** is the enantiomer of pramipexole, a dopamine agonist. During a clinical trial in amyotrophic lateral sclerosis patients, dexpramipexole unexpectedly reduced peripheral absolute eosinophil count (Dispenza and Bochner, 2018), most likely by induction of maturational arrest specific to the eosinophil lineage (Panch et al., 2018). A subsequent study to evaluate dexpramipexole as a steroid-sparing agent in hypereosinophilic syndrome showed efficacy in 40% of patients (Panch et al., 2018). Interestingly, two responders with gastrointestinal eosinophilia (a woman with esophageal and duodenal involvement and a man with gastric, duodenal and colonic involvement) showed complete resolution of tissue eosinophilia and symptom improvement at week 24 and 12, respectively. One SAE (a grade 1 squamous cell cancer of the skin in one subject) was reported, together with some minor AE in all patients, with central and/or peripheral nervous system–related symptoms such as insomnia (40%) and dizziness (30%) being the most common. Mood swings, palpitations, and skin rash were also noted in 20% of participants. No active trial is currently ongoing on EoE.

#### Mast Cells

An increased mast cell number, infiltrating all layers of the esophagus, has been consistently observed in EoE (Nicholson et al., 1997; Straumann et al., 2001; Aceves et al., 2010a; Niranjan et al., 2013).

More recent microarray analysis of EoE tissues confirmed this finding, showing upregulation of many mast cell–associated genes, such as those that encode carboxypeptidase 3A (CPA3), FcεR-I, and tryptase (TPSAB1) (Mulder et al., 2012).

Moreover, mast cell number and the expression level of mast cell proteases in mucosal biopsies of EoE patients correlate with symptom score, contrarily to eosinophil number (Otani et al., 2013).

The tissue mast cell content usually lessens in response to FEDs, topical corticosteroids (Aceves et al., 2010a) and anti-IL-5 therapy (Otani et al., 2013). However, the persistence of an elevated mast cell number despite resolution of esophageal eosinophilia has been linked to persistence of endoscopic abnormalities and symptoms (Bolton et al., 2020).

Similarly to eosinophils, mast cells produce numerous pro-inflammatory cytokines and molecules showing activity on the epithelial barrier and esophageal motility. In fact, beyond their known role in the T2 cascade, mast cells are capable of causing smooth muscle hypertrophy (Elieh Ali Komi and Bjermer, 2019) and release mediators such as tryptase, leukotrienes, prostaglandins, platelet-activating factor, tumor necrosis factor (TNF)-α, 5- hydroxytryptamine and histamine that can induce smooth muscle contraction (Goyal and Rattan, 1978).

##### Mast Cells as a Therapeutic Target

**Cromolyn sodium** or disodium cromoglicate is a mast cell stabilizer from the chromones class, able to suppress activation and degranulation by inhibiting calcium ion influx. Although animal model studies based on subcutaneous OVA sensitization followed by oral challenge showed some effects on eosinophilic and mast cell influx, collagen deposition, mast cell activation and Th2 immune response (Silva et al., 2020), a small randomized, double-blinded, placebo-controlled study of the use of viscous oral cromolyn sodium in children with EoE found no effect on eosinophilic infiltrate and symptoms (Lieberman et al., 2018).

#### Invariant Natural Killer T Cells

Invariant natural killer T cells are a subset of lymphocytes that recognize lipid and glycolipid antigens presented on CD1d molecules, can produce type 2 cytokines and have been postulated to play a pathogenic role in EoE and some other atopic diseases (Bendelac et al., 2007).

In animal models, activation of iNKT by their specific agonist is sufficient to induce EoE (Rayapudi et al., 2014).

Indeed, increased esophageal iNKTs have been repeatedly reported in EoE pediatric and adult patients, especially in the active state of the disease (Jyonouchi et al., 2013; Rayapudi et al., 2014) and in early-onset patients (Lexmond et al., 2014). This esophageal increase is associated with a reduction of peripheral blood iNKTs (Upparahalli Venkateshaiah et al., 2021). This could be explained by iNKT cells homing in the tissue via receptor CXCR6 and esophageal epithelial cells have been described as the source of CXCL16 (Rayapudi et al., 2014).

Interestingly, iNKTs from patients with active EoE expand more readily and produce more IL-13 in response to cow milk-derived sphingomyelin (Jyonouchi et al., 2013), thus suggesting a possible role of iNKT in food allergen recognition.

##### Invariant Natural Killer T Cells as a Potential Therapeutic Target

Invariant Natural Killer T cells neutralization via anti-mCD1d or anti-hVα14Jα18 antibodies protected against the development of EoE in a murine model based on intranasal peanut and *Aspergillus* sensitization (Upparahalli Venkateshaiah et al., 2021). Consequently, iNKT cell neutralization by humanized anti-CD1d and anti-Vα24Jα18 antibodies has been suggested as a potential therapy for human EoE but no studies on humans been proposed yet.

#### T Cells

T cells are an important constituent of EoE inflammatory milieu (Straumann et al., 2001; Teitelbaum et al., 2002; Blanchard, 2006; Lucendo et al., 2007). Studies on EoE biopsies describe an elevation in both CD4+ and CD8+ T cell numbers and an increase in the CD8+ T cell/CD4+ T cell ratio (Teitelbaum et al., 2002; Lucendo et al., 2007).

Th2 cells play an important role in the chemotaxis of eosinophils and initiation of the T2 cytokine cascade.

Th2 cells similar to those found in AD and EGE, producing IL-13- and IL-5 and expressing CD161 and hematopoietic prostaglandin D synthase, were detected in EoE patients (Mitson-Salazar et al., 2016).

Moreover, Th2 cells with CD154 + IL5 + phenotype were found in the peripheral blood of active EoE patients but not in the control atopic patients or inactive EoE patients. Further, these peripheral T cells could be stimulated by milk antigens in milk-induced EoE patients but not in control patients (Cianferoni et al., 2018).

In addition, a significantly higher number of stimulated CD3 + CD8 + T cells, able to produce TNF-α and interferon (IFN)-γ was described in active EoE (Sayej et al., 2016).

T cells in EoE patients are also able to express the TNF-related cytokine LIGHT (TNF superfamily member 14), which can induce an inflammatory phenotype in fibroblasts (Manresa et al., 2020).

Regulatory T cells (T_*regs*_), another subset of T lymphocytes, are critical in preventing and controlling many autoimmune and allergic diseases. The esophageal tissue of adult EoE patients shows reduced T_*regs*_ and this finding is not modified by steroid therapy (Stuck et al., 2011). In contrast, pediatric patients display a relative increase of these cells (Tantibhaedhyangkul et al., 2009; Fuentebella et al., 2010).

Recently, two cellular CD4^+^ populations called T7 (likely deregulated T_*reg*_) and T8 (likely deregulated Th2 cells), not able to suppress the adaptive response and Th2 cytokine production, were described in EoE patient’s esophageal tissue (Wen et al., 2019).

##### T Cells as a Therapeutic Target

**Azathioprine** or 6-mercaptopurine, a purine analog, has been reported to induce clinical and histological remission in steroid-refractory adult and pediatric EoE patients in some case reports (Netzer et al., 2007). Common side effects include allergic reactions, pancreatitis, bone marrow suppression, nausea and infections.

**BT-11** (piperazine-1,4-diylbis((6-(1H-benzo[d]imidazol2-yl)pyridin-2-yl)methanone) dihydrochloride), an oral, gut-restricted, small molecule that activates lanthionine synthetase C-like 2 (LANCL2) (Leber et al., 2019), has demonstrated to be able to lead to an increase in number and function of regulatory CD4 + T cells (Tregs) in Inflammatory bowel disease (IBD) and is programmed to be tested in EoE patients in a phase Ib study (NCT04835168) planned to start in January 2022.^5^

#### Epithelial Cells

The epithelium plays an important role in EoE inflammation.

Esophageal epithelial cells express toll-like receptors (Lim et al., 2009; Mulder et al., 2011) and produce pro-inflammatory cytokines and lipid mediators in response to both pathogen-associated and danger-associated molecular patterns (Straumann et al., 2001; Fillon et al., 2009; Lim et al., 2009).

Epithelial cells of EoE patients can also produce alarmins (TSLP, IL-25, and IL-33), RANTES (CCL5), a chemotactic factor for T cells, eosinophils, and basophils (Jyonouchi et al., 2013), and CXCL16 (Lexmond et al., 2014), a chemotactic factor for iNKT cells. Moreover, the esophageal epithelium is the main source of eotaxin-3 production following IL-13 stimulation (Blanchard, 2006). In addition, epithelial cells can act as non-professional antigen-presenting cells (APCs) (Mulder et al., 2011).

Therapeutic approaches are targeted at the individual cytokines and will be treated in the relative paragraphs.

#### Other Cell Types

##### Group 2 Innate Lymphoid Cells

Innate lymphoid cells (ILC2s) are tissue resident cells that express prostaglandin D2 receptor 2 (CRTH2) and, following IL-33 and TSLP signaling, can produce type 2 cytokines (Mjosberg et al., 2011; Christianson et al., 2015; Doherty et al., 2015).

A single study (Doherty et al., 2015) showed their presence in active EoE biopsies, especially in PPI unresponsive patients, as well as a correlation between ILC2 and eosinophil number.

No ILC2-targeted therapeutic approach has been found in literature.

##### Basophils

Basophils can secrete various type 2 cytokines and act as APCs (Voehringer, 2013).

An increased number of basophils has been reported by multiple studies in EoE (Noti et al., 2013; Iwakura et al., 2015). In particular, this population develops in presence of TSLP (Siracusa et al., 2011) and expresses high levels of IL-4 (Noti et al., 2013) and the IL-33R (Siracusa et al., 2011).

No basophil-targeted therapeutic approach has been found in literature.

### Soluble Immune Effectors: The Interplays

#### Cytokines

##### Interleukin-4 and Interleukin-13

Interleukin (IL)-4 and IL-13 share their alpha-chain and their receptor, but also activate specific receptors. They are known to initiate T2 immune response by inducing skewing of naive Th cells into Th2 cells, class switching to IgE production in B cells and macrophage maturation toward the M2 subpopulation, increasing dendritic cells activity, and activating and recruiting eosinophils (Blanchard et al., 2007).

These two cytokines can be produced in EoE patients by TSLP-elicited basophils, Th2 cells, Tc2 cells, iNKT, eosinophils, mast cells, activated fibroblasts and epithelial cells (Straumann et al., 2001; Lucendo et al., 2008; Zhu et al., 2010).

IL-13 and IL-4 signaling utilizes the JAK-STAT pathway, in particular STAT6 (Shukla et al., 2015), leading to eosinophilic recruitment (CCL26/eotaxin-3 gene) and downregulation of the EDC (cAPN-14 and SPINK7 genes) (Zeisberg, 2008; Kagalwalla et al., 2012).

IL-13 is currently recognized as a major effector cytokine in EoE, while IL-4 is less abundant (Blanchard et al., 2007). In a murine model, intratracheal IL-13 administration was able to induce EoE in a STAT6-dependent way, with a contribution of IL-5 and eotaxin (Mishra and Rothenberg, 2003). As a confirmation, PheWas studies have described that IL-13 polymorphisms are common in EoE (Blanchard et al., 2007, 2010). IL-13 is overexpressed in the esophageal mucosa of EoE patients (Blanchard et al., 2010), induces a gene transcript profile similar to the EoE-specific esophageal transcriptome (Kc et al., 2015), and has effects on eosinophil recruitment, esophageal barrier function, and tissue remodeling (Blanchard et al., 2007; Brightling et al., 2010; Zuo et al., 2010; Sherrill et al., 2014a).

In particular, IL-13, by inducing the expression of adhesion molecules such as VCAM-1 and ICAM-1 on blood vessels, facilitates the release of eosinophils from the bone marrow and induces the production of IL-5 by T and B cells, thus contributing to eosinophils homing and survival (Klion et al., 2020).

IL-13 also has a role in tissue remodeling: it promotes epithelial hyperplasia, collagen deposition and angiogenesis (Zuo et al., 2010) and, together with TGF-β1, activates quiescent fibroblasts to transdifferentiate into myofibroblasts and reduce the amplitude of esophageal muscle contraction (Rieder et al., 2014).

*Interleukin-4 and Interleukin-13 as Therapeutic Targets*. Two monoclonal antibodies targeting circulating IL-13 (dectrekumab and cendakimab) and a monoclonal antibody targeting the IL4/IL13 receptor (dupilumab) have been tested in patients with EoE.

**Dectrekumab** (QAX576), a fully human anti-IL-13 mAb, was the first anti-IL-13 therapy used on EoE patients. It received Orphan Drug Designation status from the FDA^6^ and EMA^7^ in 2013. A double-blind, placebo-controlled study on 23 adults showed improvement in symptoms, esophageal eosinophilia (60% improvement in treated patients versus 23% improvement in the placebo group), and disease-related transcripts. In particular, the expression of eotaxin-3, periostin and markers of mast cells and barrier function were reduced (Rothenberg et al., 2015). However, no patient achieved histological remission. Importantly, the effects of this drug (including the number of intraepithelial eosinophils and gene expression profiles) persisted for 6 months after treatment discontinuation, suggesting a potential role of this medication in combination therapies. No significant AE was reported. Nevertheless, the study of this drug in EoE has since then been discontinued.

**Cendakimab** RPC4046/CC-93538 is a recombinant, humanized, monoclonal antibody that inhibits binding of IL-13 to both IL-13Rα1 and IL-13Rα2 receptors (Tripp et al., 2017; Muir et al., 2019). Cendakimab received Orphan Drug Designation status from the FDA in 2015.^8^ A 16-week, double-blind, placebo-controlled, phase 2, multicenter study in adults with active EoE showed the ability of this drug to significantly reduce eosinophil counts, with a partial remission rate of 50% and a deep remission rate of 25% (Hirano et al., 2019). Macroscopic esophageal appearance improved as well; however symptomatic improvement was not significant. Of note, symptomatic improvement was best achieved in patients with steroid-refractory disease. AE rates were not different from the placebo group. However, four patients discontinued the drug due to dizziness, influenza-like illness, pruritus and rash, and worsening of EoE symptoms.

In a longer (52-week) open label study a prolonged symptomatic and histological improvement was initially briefly described (Dellon et al., 2019a). The recent publication (Dellon et al., 2021) reports that most patients maintained responses through week 52, with an improved symptom remission rate and an induction of remission in 36% of previously non-responding patients. The tolerability of the drug was confirmed, the most commonly reported AEs being upper respiratory tract infections, nasopharyngitis, sinusitis and headache, mainly in the induction period.

A phase 3, placebo-controlled induction and long-term study (NCT04753697) in adult and adolescent subjects is currently ongoing.^9^

**Dupilumab** is a IL4/IL13 receptor antagonist, at present approved for the treatment of severe forms of AD, asthma and nasal polyposis (Blauvelt et al., 2017). Dupixent received Orphan Drug Designation from the FDA in 2017 for the potential treatment of EoE.^10^

A recently published phase 2, double-blind, placebo-controlled RCT showed that dupilumab (600-mg loading dose followed by 300 mg weekly) induced a significant improvement in the Dysphagia Symptoms Score, an 82.6% remission rate and a 65.2% deep remission rate. Endoscopic and histological involvement, as well as esophageal compliance (measured with endoFLIP) significantly improved (Hirano et al., 2020). The most common AEs reported were injection-site reactions and nasopharyngitis. The long-term effects of dupilumab (in terms of tolerability and efficacy) are currently being studied by two phase 3 RCTs (one in adults and adolescents – NCT03633617, and one in pediatric patients – NCT04394351).^11^,^12^ A phase 2 trial (NCT03678545) is evaluating its applicability in EG and EGE^13^.

#### Interleukin-5

IL-5 is produced by Th2 cells, mast cells, eosinophils, natural killer cells, ILC2 cells and basophils. It regulates eosinophil maturation, survival and activation (O’Byrne et al., 2001) through multiple signal transduction pathways including nuclear factor kappa-light-chain-enhancer of activated B cells (NF-κB) and Janus kinase (JAK)/STAT5 modules (Roufosse, 2018).

IL-5 is overexpressed in the esophagus of patients with EoE (Straumann et al., 2001; Bullock et al., 2007; Molina-Infante et al., 2014) and correlates with esophageal eosinophilia and disease activity (Namjou et al., 2014). Blood-circulating lymphocytes producing high amounts of IL-5 and correlating with the severity of esophageal tissue eosinophilia have also been described (Bullock et al., 2007).

Finally, an association between polymorphisms in the IL5–IL13 region was also reported in a PheWAS study (Namjou et al., 2014).

##### Interleukin-5 as a Therapeutic Target

In murine models, mucosal IL-5 overexpression is able to induce EoE and IL-5 neutralization can nearly completely avoid intratracheal IL-13-induced EoE (Mishra and Rothenberg, 2003).

**Mepolizumab**, a fully humanized monoclonal IgG1 kappa anti-IL-5 antibody approved for severe asthma and hypereosinophilic syndrome, has been studied in both adult and pediatric EoE.

A first case report on three adults (Rothenberg et al., 2008) reported an improvement of symptoms, endoscopic features and esophageal eosinophil counts but absence of histologic remission.

A little and very short double blind, placebo-controlled trial in 11 adults (Stein et al., 2006) receiving two weekly intravenous infusions of 750 mg of mepolizumab and evaluated 2 weeks later, showed a reduction in esophageal infiltrate but absence of histologic remission and endoscopic or symptomatic improvement. The most common AE were headache and upper respiratory tract infection symptoms. Other reported AE were hypotension, nausea, fatigue, non-specific chest pain, and cough during infusions. No further improvements were reported after two additional infusions of 1500 mg of mepolizumab at 4-week intervals (Straumann et al., 2010b).

A subsequent non-placebo-controlled trial involving 3 different doses on 59 pediatric patients (Assa’ad et al., 2011) reported, after 12 weeks, a significant reduction in epithelial eosinophil counts, with 31.6% of children reaching remission, and only 8.8% achieving deep remission. There was no symptoms improvement and at week 24, with no additional mepolizumab doses, eosinophilia increased in all arms. The most common AEs were vomiting, diarrhea and upper abdominal pain.

A new multi-center, randomized, double-blind, parallel-arm, placebo-controlled trial (NCT03656380) in adults and adolescents with mepolizumab 300 mg subcutaneously monthly for 3 months or mepolizumab 100 mg SQ monthly is currently ongoing.^14^

**Reslizumab** is a humanized IgG4 kappa monoclonal antibody that binds to human IL-5, approved for severe eosinophilic asthma (Castro et al., 2015). It received Orphan Drug Designation status from the FDA in 2007.^15^ A double blind, placebo-controlled trial on 227 patients receiving four intravenous infusions of 1, 2, or 3 mg/kg of reslizumab every 4 weeks showed a reduction of the eosinophilic infiltrate but a disease remission in only 4.4% of patients (Spergel et al., 2012b). No reported AE resulted significantly different from the placebo group. However, a subsequent study reporting the follow-up data of 12 patients undergoing the open-label extension of this trial or receiving reslizumab as a compassionate use for up to 9 years, reported that 92% of the children showed a reduction in eosinophil count to less than 5/hpf and a significant symptomatic improvement (Markowitz et al., 2018).

#### Interleukin-15

IL-15, produced predominantly by monocytes, macrophages, and dendritic cells, has a structural similarity to IL-2, with which it shares the ability to stimulate the proliferation and differentiation of activated T cells (Anderson et al., 1995). In addition, IL-15 is required for NK cells and some T cells survival and activation (Kennedy and Park, 1996). IL-15 has also been shown to support type 2 allergic inflammatory responses: it can induce murine mast cells to produce IL-4 through the STAT6 pathway and esophageal epithelial cells to increase the expression of eotaxin (Tagaya et al., 1996; Masuda et al., 2000). Moreover, IL-15 can induce skewing of CD4 + T and iNKT cells (Lucendo et al., 2008) and induce the synthesis of IL-5 and IL-13 (Mishra et al., 2008; Blanchard et al., 2010).

IL-15 and its receptor IL-15Ra are elevated in esophageal tissue samples of patients with EoE, as well as in an intranasal *Aspergillus*-induced murine model of EoE, and correlate with esophageal eosinophilia (Zhu et al., 2010). Interestingly, peripheral blood IL-15Ra mRNA levels are reduced, seemingly indicating that most of the cells expressing IL-15Rα moved to the tissue (Upparahalli Venkateshaiah et al., 2021).

*Interleukin-15 as a Therapeutic Target*. IL-15-deficient mice show a significantly reduced esophageal eosinophilia when challenged with intranasal *Aspergillus fumigatus* (Zhu et al., 2010; Venkateshaiah et al., 2019).

**CALY-002** is a first-in-class humanized monoclonal antibody inhibiting IL-15. It received Orphan Drug Designation from EMA in 2016^16^ and from the FDA in 2018^17^ for the potential treatment of EoE. A multi-site, randomized, placebo-controlled study (NCT04593251) in celiac patients that includes an open label multiple dose expansion cohort in participants with EoE is currently ongoing.^18^

##### Interleukin-18

IL-18 shares structural similarities with IL-1 (Nakanishi et al., 2001). Myeloid cells constitutively produce the inactivated version of this cytokine (proIL-18), and the conversion into its bioactive form (IL-18) occurs only upon inflammasome activation, through the enzymatic activity of caspase-1. In the extracellular space, IL-18 exerts its inflammatory activity via binding to its receptors, IL-18Rα and IL-18Rβ (Van Gorp et al., 2019). Initially it was thought that this cytokine mainly played a role in inducing Th1 differentiation, however it is also known to mediate T2 responses as well. Some IL-18 polymorphisms have been linked to a decreased risk of atopy, and IL-18 was shown to induce T2 inflammation in animal models (Tsutsui et al., 2004; Cheng et al., 2014). This cytokine is indeed able to induce iNKT cell production of the key T2 cytokines IL-5 and IL-13 (Niranjan et al., 2015).

It appears that the esophageal mucosa of EoE patients is characterized by a high expression of the IL-18 mRNA, as well as higher distribution of IL-18Rα (Niranjan et al., 2015).

These findings were confirmed in a murine model of *Aspergillus* and peanut-induced EoE (Dutt et al., 2015), where high levels of circulating IL-18 and esophageal IL-18Rα mRNA were detected. Moreover, this study confirmed that IL-18Rα was expressed by esophageal iNKT cells and that intranasal administration of IL-18 resulted in a time- and dose-dependent esophageal mast cell and eosinophilic inflammation. IL-18 overexpression was also able to induce eosinophilic and mast cell infiltration of the esophagus and increased collagen deposition in the epithelial mucosa, lamina propria, and muscularis mucosa.

*Interleukin-18 as a Therapeutic Target*. Based on the previously cited findings, IL-18 or IL8Rα have been proposed as therapeutic targets for EoE by many authors (Dutt et al., 2015; Niranjan et al., 2015; Shukla et al., 2015).

Indeed, **tadekinig alfa**, a human recombinant IL-18 binding protein (IL-18BP) (Gabay et al., 2018) is being actively studied in Still Disease and other autoinflammatory diseases (NCT03113760) and **GSK1070806,** a humanized IgG1/kappa antibody directed against the soluble cytokine interleukin-18 (IL-18), is starting to be employed in type 2 Diabetes Mellitus (McKie et al., 2016), renal transplant recipient (Wlodek et al., 2021) and Crohn’s disease.^19^ However, their use in EoE patients has not yet been studied.

##### Interleukin-33

IL-33 is an alarmin cytokine of the IL-1 family, released in response to injury. IL-33 binds to a receptor complex of IL-1 receptor-like 1 (IL1RL1, also known as ST2) and IL-1 receptor accessory protein (IL1RAcP). It is constitutively produced by endothelial cells, epithelial cells, keratinocytes and dendritic cells (Geering et al., 2013) and can also be expressed by other immune cells upon activation, including eosinophils, mast cells, macrophages, dendritic cells (DCs), B cells and monocytes (Johnston and Bryce, 2017). IL-33 can activate the STAT6 pathway, increases IL-5 production and is required for eosinophil survival (Johnston et al., 1950). Additionally, it induces cytokines release from mast cells and Th2 cells and induces the production of IL-1 beta, IL-4, IL-5, IL-6, IL-8, IL-13 and GM-CSF from basophils (He et al., 2013).

IL-33 expression is increased in pediatric EoE (Judd et al., 2016) and appears to be expressed by both the endothelium and the basal layer of the epithelium by an undifferentiated, non-dividing esophageal epithelial cell population (Judd et al., 2016; Travers et al., 2016). Moreover, an increased expression of ST2 (IL-33R) has been described in other studies, especially in basophils (Siracusa et al., 2011; Venturelli et al., 2016).

IL-33 is able to promote EoE development in mice (Judd et al., 2016) and a murine model of EoE based on epicutaneous OVA sensitization followed by intranasal OVA challenge, revealed that the IL-33–ST2 axis, together with the expression of ST2 on basophils, were required to induce EoE (Venturelli et al., 2016).

*Interleukin-33 as a Therapeutic Target*. ST2-deficient mice do not develop EoE (Venturelli et al., 2016), thus suggesting that drugs acting on the IL-33-ST2 axis might be efficacious in EoE.

Currently, two drugs are being investigated in other T2- inflammation-related conditions.

**Etokimab** (ANB020), an IgG1 anti-IL-33 monoclonal antibody is being investigated in severe eosinophilic asthma,^20^ food allergy (Chinthrajah et al., 2019), and AD (Chen et al., 2019) with promising results.

**Astegolimab** (MSTT1041A/AMG 282), a human IgG2 monoclonal antibody that selectively inhibits ST2, reduced asthmatic exacerbation rate in a broad population of patients (Kelsen et al., 2021). However, no study has been announced in EoE.

##### Transforming Growth Factor Beta

Transforming growth factor beta regulates epithelial growth and tissue remodeling and has complex roles in cell differentiation, proliferation, apoptosis and in immune regulation. It is produced by various cells, including mast cells, eosinophils, fibroblasts and epithelial cells.

An elevated TGF-β expression has repeatedly been found in esophageal biopsy samples of patients with active EoE (Aceves et al., 2007; Mishra et al., 2008; Straumann et al., 2010a), especially in mast cells (Aceves et al., 2010a). Also other TGF-beta-induced factors, such as plasminogen activator inhibitor-1 (PAI-1) (Rawson et al., 2016) and Thrombospondin-1 (TSP-1) (Hsieh et al., 2021) are significantly increased in the EoE esophageal epithelium and are associated with fibrosis and collagen deposition.

Indeed, multiple studies have shown a major role of TGF-β1 in esophageal remodeling through stimulation of fibroblast secretion of extracellular matrix and expression of periostin and smooth muscle actin (Muir et al., 2016; Rawson et al., 2016; Hsieh et al., 2021), activation of transdifferentiation into myofibroblasts (Aceves et al., 2010a) and EMTs (Kagalwalla et al., 2012; Rieder et al., 2014).

Moreover, TGF-β1 can increase esophageal smooth muscle contractility (Aceves et al., 2010a) by preventing the uptake of cytosolic calcium through the action of phospholamban (PLN) (Beppu et al., 2014).

TGF-β1 exposure also leads to decreased epithelial barrier function. TGF-β can induce epithelial cells apoptosis through p38 mitogen–activated protein kinase (MAPK)– signaling pathway, reduce the expression of the tight-junction molecule claudin-7 (CLDN7) (Nguyen et al., 2018) and induce matrix metalloproteinase expression in esophageal epithelial cells.

A relationship between TGF-β and EoE pathogenesis was also suggested by some inconstant genetic associations: a polymorphism (SNP C509T) in the TGFβ1 promoter on chromosome 19q13 was associated with an increase of TGFβ1 and tryptase and with increased remodeling, especially in the presence of concurrent food sensitization and steroid unresponsiveness (Aceves et al., 2010b; Rawson et al., 2015; Duong et al., 2020). Moreover, the repeatedly reported association between EoE and polymorphisms in locus 11q13, attributed to EMSY by most authors [see section “Tumor necrosis factor-α and Interferon-γ”], was recently attributed by Kottyan et al. (2021) to the near encoded protein LRRC32 (leucine-rich repeat-containing protein 32; also known as GARP), a TGF-β binding protein with a role in latent surface expression and signaling of TGF-β and highly expressed in activated forkhead box P3 (FOXP3) + T regulatory cells (D’Mello et al., 2016). Interestingly, there is also a high rate association of EoE with Loeys-Dietz syndrome, caused by gain-of-function mutations in the TGF-β receptors (Frischmeyer-Guerrerio et al., 2013).

*Transforming Growth Factor Beta as a Therapeutic Target*. The evidence that Smad3 (TGF-β signaling pathway), -deficient EoE mice show reduced esophageal fibrosis and angiogenesis (Cho et al., 2014) has induced the theory that anti-TGF-β therapy could be used to prevent fibrosis and remodeling in EoE patients.

Indeed, the use of neutralizing antibodies to TGF-β has shown to prevent organ fibrosis in murine models of different diseases (McCormick et al., 1994, 1999; Ziyadeh et al., 2000; Fukasawa et al., 2004) and *in vitro* (McMillan et al., 2005). However, in different murine models of allergic airways disease, treatment with anti–TGF-b led to contrasting results (McMillan et al., 2005; Fattouh et al., 2008).

In humans, **Fresolimumab**, a monoclonal anti–TGF-β antibody, decreased biomarkers and reversed skin fibrosis in systemic sclerosis patients (Rice et al., 2015), however no studies have been carried out in EoE patients.

In addition to targeted therapy, several commonly used drugs have demonstrated anti-TGF-β activity and thus constitute a possible anti-fibrotic therapeutic strategy.

**Losartan**, an angiotensin II receptor blocker, has shown anti-fibrotic activity by blocking angiotensin II induction of TGF-β in numerous animal models and in some inconstant and small human trials of organ fibrosis (Couluris et al., 2012; Salama et al., 2016). A preliminary, open-label trial (NCT01808196) of losartan potassium in pediatric EoE patients with or without a CTD^21^ and a phase 2 trial (NCT03029091) with losartan assessing this drug’s potential to achieve endoscopic, histological, and symptomatic improvement in 14 pediatric and adult EoE patients with or without CTDs at 16 weeks^22^ have been completed respectively in February 2015 and in September 2020 but the results have not been published. The results available on Clinicaltrials.gov, however, show very modest effects on eosinophilic infiltrate, remission, histologic and endoscopic scores and symptoms. No data is available on fibrosis or esophageal compliance.

**Thiazolidinediones** rosiglitazone and pioglitazone, two PPARγ agonists, significantly inhibited TGF-β1-induced mRNA expression of several remodeling-related genes *in vitro* in EoE esophageal fibroblasts with a dose-dependent effect, disrupting TGF-ß-Smad signal transduction (Nhu et al., 2020).

**Tranilast** is an anthranilic acid derivative that prevents mast cell degranulation, in use in Japan for the treatment of allergic rhinitis, asthma and AD. In an *in vitro* study, tranilast prevented the secretion of TGF-ß, and blocked TGF-ß-induced Smad2 and ERK1 activation (Platten et al., 2001).

##### Thymic Stromal Lymphopoietin

Thymic stromal lymphopoietin and its receptor promote Th2 differentiation and dendritic cells activation (Ziegler, 2010).

It is produced by epithelial cells, macrophages, mast cells and dendritic cells (Geering et al., 2013) in response to cytokines (Bogiatzi et al., 2007), noxious substances (Smelter et al., 2010), and mechanical stress (Oyoshi et al., 2010).

It also has a broad variety of cell targets, including eosinophils (Wong et al., 2010), mast cells (Allakhverdi et al., 2007), and basophils (Siracusa et al., 2011).

An increased TSLP expression has been repeatedly reported in the esophageal tissue of patients with EoE (Blanchard, 2006; Rothenberg et al., 2010).

As a confirmation of the major role of this cytokine in EoE, the TSLP locus (5q22) is highly associated with EoE in multiple GWAS studies (Sherrill et al., 2010; Kottyan et al., 2014, 2021; Namjou et al., 2014; Sleiman et al., 2014). In particular, a TSLP single nucleotide polymorphism (SNP), that results in increased TSLP expression, correlates with EoE risk and increased basophil numbers (Rothenberg et al., 2010; Noti et al., 2013).

Moreover, a single nucleotide variation (rs36133495) in the cytokine receptor-like factor 2 (CRLF2) gene, that codes for the receptor of TSLP and is located on the pseudoautosomal region of the sex chromosomes, was found to be associated to EoE in males and is thus the first molecular mechanism that associate EoE to its male predominance (Sherrill and Rothenberg, 2011; Holvoet et al., 2016).

*Thymic Stromal Lymphopoietin as Therapeutic Target*. Targeting TSLP decreased eosinophilia and total immune cell infiltration in a murine model of EoE induced by epicutaneous OVA or peanut sensitization followed by oral challenge (Noti et al., 2013).

**Tezepelumab** (AMG 157) is a fully human anti-TSLP antibody with potential use in uncontrolled asthma (Corren et al., 2017). On October 8th, 2021 Tezepelumab was granted Orphan Drug Designation (ODD) in the US by the FDA for the treatment of EoE.^23^

##### Eotaxin-3 or C-C Motif Chemokine Ligand 26 (CCL26)

Eotaxins, a subfamily of chemokines, are critical for the chemotaxis of eosinophils into tissues. They are mainly produced by epithelial cells, but can also be produced by activated eosinophils, mast cells and fibroblasts (Forssmann et al., 1997). The eotaxin subfamily consists of three different molecules. Eotaxin-1 attracts eosinophils in the gastrointestinal tract (except for the esophagus), eotaxin-2 is mostly expressed in the lung, while eotaxin-3 is found in lungs and gastrointestinal tract (Forssmann et al., 1997).

They are upregulated by IL-4 and IL-13 via STAT6 and signal exclusively through receptor CCR3, which is expressed predominantly on eosinophils and mast cells, but is also found on basophils Th2 cells (Rothenberg et al., 1995; Forssmann et al., 1997; Kitaura et al., 1999). Its activation causes an increase of cell adhesion molecules and the production of T2 cytokines such as IL-13.

Eotaxin-3 seems to be less potent and to be expressed later than eotaxin 1 and 2 and is seems to be involved in prolonged eosinophil recruitment (Ravensberg et al., 2005).

Eotaxin-3 is the most expressed cytokine in all EoE patients (Blanchard et al., 2006; Sherrill et al., 2014b). In particular, it had the highest differential value between patients and controls and its expression strongly correlates with eosinophil and mast cell infiltrate (Blanchard et al., 2006).

Additionally, an increased expression of PARP14, a transcriptional cofactor that facilitates CCL26 transcription, was also described in EoE pediatric patients. CCL26 expression strongly correlates with PARP14 expression (Krishnamurthy et al., 2014).

Finally, a single-nucleotide polymorphism on the eotaxin-3 gene (chromosome 7q11) has been associated with increased eotaxin-3 levels and an increased EoE risk, possibly through an enhancement of the stability of the eotaxin-3 mRNA (Blanchard et al., 2006; Romano et al., 2014; Moawad et al., 2015).

*Eotaxin as a Therapeutic Target*. CCL26 expression is downregulated upon treatment with topical steroids (Blanchard, 2006) and PPIs (Zhang et al., 2012).

Also CCR3 has been identified as a promising target for novel therapies directed to eosinophilic diseases, and thus, to have a possible role in EoE therapy.

**GW766994**, an oral small-molecule selective competitive antagonist of CCR3, has been investigated in asthma with no significant results (Neighbour et al., 2014).

More recently, **R321,** a peptide-based CCR3 antagonist with a different mechanism of action was described (Grozdanovic et al., 2019). In mouse models of acute allergic airways disease R321 blocked eosinophil recruitment into the blood, lungs, and airways and prevented airway hyperresponsiveness. No further studies on this molecule are currently available in literature.

At present, no studies in EoE with these drugs have been proposed.

##### Tumor Necrosis Factor-α and Interferon-γ

Even if it is widely recognized that EoE pathogenesis is mainly orchestrated by T2 responses, Th1-related cytokines, including TNF–α and IFN-γ are found to be increased in EoE tissue specimens (Straumann et al., 2001; Gupta et al., 2006; Ruffner et al., 2021).

It was described that CD8 + T cells are a source of these cytokines in active disease settings, but not during remission or in control groups (Sayej et al., 2016). Moreover, circulating CD4 + T cells from pediatric EoE patients were able to produce interferon upon stimulation with EoE-causal allergens (Ruffner et al., 2021).

TNF-α derived from dendritic cells, eosinophils, mast cells and T cells is able to induce epithelial cell contraction, migration, and collagen secretion, as well as the expression of adhesion molecules on endothelial cells (Straumann et al., 2001; Rieder et al., 2014; Upparahalli Venkateshaiah et al., 2021). These aspects may link this cytokine to tissue remodeling and angiogenesis in EoE patients. Moreover, fibroblast-derived TNF-α can stimulate epithelial expression of lysyl oxidase (LOX), a collagen cross-linking enzyme, through activation of nuclear factor κB and TGF-β–mediated signaling. LOX upregulation has been linked to EoE complications, including fibrostenotic alterations (Kasagi et al., 2019).

Genetically, an association between EoE risk and polymorphisms in gene c11orf30 has been described (Sleiman et al., 2014). This gene encodes for EMSY, a transcriptional regulator recently found to be involved as a critical mediator of a novel Akt-dependent mechanism exploited by IFN and other growth factors to regulate interferon-stimulated genes (Ezell et al., 2012). EMSY expression was also found to be significantly enhanced in active EoE compared to controls (Kottyan et al., 2021). Variants at the c11orf30 locus have also been associated with seasonal allergic rhinitis (Ramasamy et al., 2011), ulcerative colitis (Anderson et al., 2011), Crohn’s disease (Barrett et al., 2008), AD (Esparza-Gordillo et al., 2009; Hirota et al., 2012), asthma (Ferreira et al., 2011), and allergic sensitization (Bønnelykke et al., 2013; Amaral et al., 2015).

*Tumor Necrosis Factor-α and Interferon-γ as a Therapeutic Target*. **Infliximab**, a chimeric IgG1 monoclonal antibody that inhibits TNF-α, was studied in a short pilot study on three adult patients refractory to standard treatments (Straumann et al., 2008). An infliximab dose of 5 mg/kg at weeks 0 and 2 failed to cause a significant decrease in esophageal eosinophil counts nor in symptoms. However, the short treatment period did not consent to assess efficacy on remodeling.

No therapeutic approaches involving IFN-γ are available in literature.

#### Other Soluble Effectors

##### Eicosanoids

Prostaglandin D2 (PGD2) has an established role in allergic diseases such as asthma. It is produced by mast cells (He et al., 2013) and recruits Th2 cells, eosinophils, and basophils through receptor CRTH2 (Pettipher et al., 2007). Moreover, it mediates vasodilatation and the increase of vascular permeability (He et al., 2013). PGD2 injection in the esophagus leads to an increase of eosinophil infiltration in esophageal epithelium at the injection site (Zhang et al., 2014), and prostaglandin D synthase is expressed in adaptive CD4^+^ effector memory Th2 cells in EoE (Mitson-Salazar et al., 2016).

Other eicosanoids, such as leukotriene D4, prostaglandin F2 alpha and thromboxane B2 appear to have a role in contraction of esophageal muscle (Daniel et al., 1979; Kim et al., 1998; Rosenberg et al., 2013).

*Eicosanoids as a Therapeutic Target*. Prostaglandin inhibition was able to protect against EoE-like inflammation in an animal model (guinea pig) based on intraperitoneal OVA sensitization followed by aerosol challenge (Zhang et al., 2014).

**Timapiprant** (OC000459/CHF 6532), a CRTH2 inhibitor, was studied in a double-blind, placebo-controlled RCT of 26 adults with refractory EoE for 8 weeks (Straumann et al., 2013). The study reported a significant decrease in eosinophilic infiltrate and symptoms with a trend toward normalization of endoscopic features, but absence of remission. No significant AEs were reported.

**Montelukast,** a competitive, selective leukotriene D4 receptor antagonist that has been used since long time in asthma treatment, was evaluated as a possible adjunctive treatment to maintain remission in EoE patients after steroid therapy with no significant results and no significant AEs (Lucendo et al., 2011b; Alexander et al., 2017).

##### Histamine

The histamine receptors HR1, HR2, and HR4 have been reported to be highly expressed the esophagus of patients with active EoE, in particular by epithelial eosinophils (Merves et al., 2015).

Histamine is produced by mast cells and induces vasodilation, microvascular leakage, dendritic cells polarization toward T2, eosinophilic homing, production of IL-6 in mast cells and fibroblast and endothelial proliferation (He et al., 2013). Moreover, histamine could also play a role in smooth muscle contraction (Goyal and Rattan, 1978). However, no further studies have assessed histamine role in EoE.

*Histamine as a Therapeutic Target*. Up to date, no clinical trial or case report was published on the use of antihistamines in EoE.

A phase II, randomized, placebo-controlled study (NCT04248712) evaluating the efficacy of antihistamines (the anti-H2 famotidine and anti H1 loratadine) in the treatment of EoE (the ATEE Study) is currently ongoing.^24^

##### Immunoglobulins E and Immunoglobulins G

Local IgE production by B cells (Vicario et al., 2009) and an increase of FcεRI-positive cells (namely Langherans cells) (Yen et al., 2010) have been described in EoE.

Also, an increased level of IgG4 in homogenates of esophageal tissues, granular extracellular IgG4 deposits in lamina propria in biopsy specimens and an increased total serum level of IgG4 with increased serum levels of IgG4 that reacted with milk, wheat, egg, and nuts have been reported (Clayton et al., 2014; Zukerberg et al., 2016; Schuyler et al., 2017, 2018; Wilson et al., 2017; Rosenberg et al., 2018; Weidlich et al., 2020).

However, other studies collecting IgG4 directly from the esophageal mucosa showed no differences in the levels of food-specific IgG4 between patients and controls (Ramaswamy et al., 2019) and studies evaluating the efficacy of serum or mucosal IgE or IgG-directed tests in the identification of causal food allergens yielded disappointing results.

Moreover, B cell-deficient mice are still able to develop EoE (Mishra et al., 2007), thus indicating a non-crucial role of humoral immunity in EoE pathogenesis.

*IgE and IgG as a Therapeutic Target*. **Omalizumab**, a monoclonal antibody that binds to free serum IgE and prevents it from binding to high-affinity receptor Fche identification of causal food allergens yielded disappointibronchial asthma and chronic urticaria, has been investigated in EoE with scarce results. In particular, some case reports (Rocha et al., 2011; Arasi et al., 2016) and a randomized double-blind placebo-controlled study on adults and children (Clayton et al., 2014) showed no significant histological and clinical improvements, while an open-label single-arm trial demonstrated endoscopic and histological remission in 33% of the subjects (Loizou et al., 2015).

No other therapy approach directed to humoral immunity has been found in literature.

##### Periostin

Periostin is an extracellular matrix (ECM) protein produced by esophageal fibroblasts and epithelial cells in response to TGF-β and IL-13 (Nguyen et al., 2016), that supports adhesion and migration eosinophils and regulates extracellular matrix deposition (Conway and Molkentin, 2008; Snider et al., 2008). Its expression has been reported to be highly upregulated (approximately 52-fold) in biopsies from patients with EoE (Politi et al., 2016) but not in serum (Dellon et al., 2016).

*Periostin as a Therapeutic Target*. Periostin has been shown to be reduced by diet and steroid therapy in EoE (Politi et al., 2016). Up to date, no periostin-specific therapy has been proposed for EoE. Recently, a new peptide antagonist has been developed and tested *in vitro* in the oncologic field (Oo et al., 2021).

##### Sphingosine-1-Phosphate

Sphingosine-1-phosphate is a blood borne lipid mediator derived from sphingosine which regulates eosinophils recruitment, migration of natural killer cells, mast cells activation, lymphocyte trafficking, Th17 cell polarization and dendritic cell differentiation (Sugita et al., 2010; Cohen et al., 2019). A dysregulation of S1PRs has been found in some immunological diseases, including asthma (Sun et al., 2010), but the role of S1P in EoE has not yet been studied.

*Sphingosine-1-Phosphate as a Therapeutic Target*. Several selective sphingosine-1-phosphate receptor (SP1R) modulators have yielded promising results in other immune-mediated diseases like multiple sclerosis, psoriasis, and IBD (Vaclavkova et al., 2014; Sandborn et al., 2016, 2020; Pérez-Jeldres et al., 2019).

**Etrasimod** (APD334), a SP1R modulator, has received Orphan Drug Designation status from the FDA in June 2021^25^ and is currently being evaluated in EoE in a phase 2b, randomized, double-blind, placebo-controlled trial (NCT04682639).^26^

#### Surface Proteins

##### α4β7 Integrin

α4β7 integrin is expressed on T lymphocytes, eosinophils, and natural killer cells surface (Soler et al., 2009; Wyant et al., 2016) and is gut-specific (Cherry et al., 2015).

Expression of both α4β7 and α4β1 by eosinophils has been described (Berlin et al., 1993; Cook et al., 1995), but β7 seems to be more important homing eosinophils into the GI tract^]^ through its receptor, MAdCAM-1, expressed in high endothelial venules of lymph nodes and in the lamina propria of intestine. In murine IBD models, blockade of α4β7 prevents GI eosinophilic recruitment (Picker and Butcher, 1992; Berlin et al., 1993; Gonzalo et al., 1998; Brandt et al., 2006).

This integrin appears to be also expressed on EoE Th2 cells (Wen et al., 2019). Moreover, studies suggest that Cadherin 26 promotes α4β7-mediated cell adhesion via direct interactions with α4 and β7 subunits (Caldwell et al., 2017).

*α4β7 as a Therapeutic Target*. Integrins represent a therapeutic target in some inflammatory conditions as IBDs and rheumatoid arthritis. No clinical trial has been conducted in EoE patients, but some case reports have been published.

**Natalizumab**, a humanized monoclonal antibody that binds to the α4 integrin in both α4β1 and α4β7 integrins and has demonstrated efficacy in multiple sclerosis (Tramacere et al., 2015) and Crohn’s disease (MacDonald and McDonald, 2007), has been reported to lead to complete resolution of EoE not responsive to PPIs, swallowed corticosteroids and 6FED in a woman treated for multiple sclerosis, with persistence of clinical and histological remission after 3 years of treatment (Beales, 2019).

**Vedolizumab**, an antibody that specifically blocks α4β7, has led to complete resolution of EoE in 2 patients receiving treatment for Crohn disease (Nhu et al., 2018; Taft and Mutlu, 2018).

This finding is associated with the description of vedolizumab as an effective rescue therapy for eosinophilic gastroenteritis in small case series (Kim H. P. et al., 2018; Grandinetti et al., 2019).

##### Siglec-8

Sialic acid-binding Ig-like lectins (Siglecs) are found on the membrane of many immune cells. They are involved in cell signaling. Siglec 8, expressed on eosinophils, mast cells, and basophils, controls eosinophil apoptosis, inhibits mast cell degranulation, and reverses tissue remodeling (Kiwamoto et al., 2012).

*Siglec-8 as a Therapeutic Target*. In a murine model of EoE based on OVA intraperitoneal sensitization and subsequent intraesophageal challenge, the administration of anti-Siglec monoclonal antibodies has significantly reduced esophageal, circulating and bone marrow eosinophils and decreased remodeling processes (Rubinstein et al., 2011). Similar results have been obtained on eosinophilic and mast cells infiltrate and on inflammatory mediators in a murine model of EGE (Youngblood et al., 2019).

**Antolimab/lirentelimab** (AK002), a humanized non-fucosylated IgG1 monoclonal antibody directed against Siglec-8, was recently evaluated in a randomized, phase 2, placebo-controlled study in adult patients with EG and/or EGE (Dellon et al., 2020), showing a 95% mean reduction of tissue eosinophilia and a clinic-histological response in 69% of patients. Of note, a subanalysis on 23 patients presenting with an associated esophageal eosinophilia reported a significant reduction of the esophageal eosinophilic infiltrate with a high esophageal remission rate and a dysphagia score reduction. The most significant AEs reported were infusion-related reactions, of which one severe, and lymphopenia with no clinical consequences. Antolimab received Orphan Drug Designation status from the FDA in 2019.^27^ A phase 2/3, multicenter, randomized, double-blind, placebo-controlled study (NCT04322708) to evaluate the efficacy and safety in adult and adolescent EoE patients^28^ is currently ongoing.

#### Transcription Factors

##### The Signal Transducer and Activator of Transcription 6 Pathway

The Signal Transducer and Activator of Transcription 6 (STAT6) pathway is pivotal in the development of EoE in intranasal *Aspergillus*-based murine models (Mishra and Rothenberg, 2003).

IL-4 and IL-13, together with IL-3, IL-15, IL-33, IFNα and platelet-derived growth factor-PDGF, can activate STAT6 through the action of Janus family (JAKs) tyrosine kinases (Huang et al., 2020). It acts as a Th2-inducing transcriptional activator (Goenka and Kaplan, 2011) and is implicated in the immunity to helminths and in the pathophysiology of various allergic conditions (Karpathiou et al., 2021).

Its effects are varied. In T cells, it decreases the expression of CDKN1B reducing proliferation; it induces Th2 differentiation and is needed for IL-9 secretion. In B cells, STAT6 induces IgE and IgG1 immunoglobulin class switching. In macrophages, it promotes differentiation to M2 and the expression of the major histocompatibility complex (MHC) class II (Goenka and Kaplan, 2011). In esophageal epithelial cells, the activation of STAT6 results in eotaxin-3 expression. In fibroblasts, it induces periostin expression (Blanchard et al., 2005).

Additionally, STAT6-mediated (Miller et al., 2019) activation of CAPN14 and SPIK7 genes (Pesek and Gupta, 2019) has been identified as the mechanism by which IL-13 downregulates the EDC and thus its effects on barrier function (Blanchard et al., 2010; Sherrill et al., 2014a; Kleuskens et al., 2021).

STAT6 polymorphisms on locus 12q13, previously associated with serum IgE levels (Granada et al., 2012), allergic sensitization (Bønnelykke et al., 2013), and asthma (Qian et al., 2014), have also been reported to have a major genetic association in EoE independent of sensitization (Sleiman et al., 2014) but a subsequent meta-analysis did not confirm the finding (Kottyan et al., 2021).

Recently, STAT6 variants were associated with relapse of EoE in pediatric patients receiving long-term PPI therapy (Mougey et al., 2021).

*The STAT6 Pathway as a Therapeutic Target*. STAT6-deficient mice do not develop EoE (Blanchard et al., 2007).

**PPIs** are an established first-line therapy for EoE, with a reported efficacy in 50% of pediatric and adult patients (Lucendo et al., 2016; Laserna-Mendieta et al., 2020; Navarro et al., 2021). Beyond their blocking effect of gastric acid secretion, PPIs have been demonstrated to be able to downregulate the esophageal gene expression of eotaxin-3, IL-5, and IL-13 similarly to topical steroids (Molina-Infante et al., 2014) and to inhibit ICAM-1 and VCAM-1 expression and enhance barrier function (Spergel et al., 2018). Indeed, *in vitro* omeprazole appears to block the chromatin remodeling that is necessary for STAT6 -mediated transcription of the eotaxin-3 gene (Zhang et al., 2012; Cheng et al., 2013). Common AEs, mainly derived from prolonged use, comprehend gastrointestinal infections, decreased vitamin B12 absorption, hypomagnesemia and increased risk for bone fractures.

**JAKSTAT6 pathway inhibitors** (ruxolitinib, AS1517499, leflunomide) have demonstrated *in vitro* ability to inhibit IL-13 induced eotaxin-3 expression in esophageal epithelial cells similar to PPIs (Cheng et al., 2013). Moreover, this effect was confirmed in esophageal fibroblasts, suggesting a role for JAK inhibitors in EoE treatment (Cheng et al., 2016).

**Tofacitinib**, a JAK inhibitor that showed some preliminary results in other eosinophilic diseases, as hypereosinophilic syndrome (King et al., 2017), bronchial asthma (Younis et al., 2019), and eosinophilic fasciitis (Kim S. R. et al., 2018; Cao et al., 2019), has recently been reported to induce clinical and endoscopic remission in a patient with refractory EoE (Mendoza Alvarez et al., 2019).

#### Nuclear Receptors

##### Peroxisome Proliferator-Activated Receptor γ

Peroxisome Proliferator-Activated Receptor γ (PPARγ) is a nuclear receptor that plays a crucial role in adipocyte physiology. Emerging evidence suggests that this molecule also play a role in macrophage and dendritic cells differentiation and polarization to M2 phenotype, lipid metabolism control in multiple immune cells and has an immune-modulatory role consisting in inhibition of Th1 and Th17 type inflammation, while its role on Th2 inflammation is still contradictory (Hernandez-Quiles et al., 2021). The IL4/STAT6 pathway stimulates PPARγ expression, and both IL-13 and STAT6 facilitate signaling through PPARγ (Huang et al., 1999). This nuclear receptor is able to regulate genes involved in lipid transport and metabolism (including the class B scavenger receptor CD36, FABP4, LXRA, and PGAR) in monocytes, macrophages and dendritic cells. Moreover, PPARγ may also play a role in lipid antigen presentation to T cells, by increasing CD1d expression in dendritic cells, making these cells more prone to activate iNKT cells (Szatmari et al., 2004). On the other side of the coin, PPARγ also appears to be linked in the downregulation of T2 cytokines in T cells, interacting with NFAT (Chung et al., 2003) and in the inhibition of mast cell maturation and activation (Zhang et al., 2017) showing a protective role in murine models of asthma and allergic rhinitis (Hammad et al., 2004; Wang et al., 2011).

In EoE, PPAR-γ-positive CD4± T cells were recently identified (Wen et al., 2019) and a study (Nhu et al., 2020) demonstrated a higher expression of PPAR-γ mRNA in EoE-derived fibroblasts, upregulated by IL-4 stimulation, and that expression of PPAR-γ was detectable in the epithelium and subepithelial lamina propria of active EoE but not in the normal esophagus.

*Peroxisome Proliferator-Activated Receptor γ as a Therapeutic Target*. Thiazolidinediones (TZDs), a class of antihyperglycemic drugs able to activate PPAR-γ, that include rosiglitazone and pioglitazone (Lehmann et al., 1995) have been proposed as potential drugs to target fibrosis in EoE, also for the ability to act mainly at the level of damaged tissue, sparing healthy areas. The TZDs preferentially exert antifibrotic effects in TGF-β1-activated EoE fibroblasts. In an *in vitro* study (Nhu et al., 2020) rosiglitazone and pioglitazone strongly inhibited TGF-β1-mediated synthesis of α-sma, collagen-1α1, and ctgf in EoE fibroblasts, in a dose-dependent fashion, while failing to do so in fibroblasts from healthy controls.

## Discussion

Eosinophilic esophagitis pathophysiology is complex and involves a still poorly understood net of genetic predisposition, mainly involving epithelial barrier and T2-related genes, and external factors that initiate a type 2 inflammatory cascade in which both innate and adaptive immunity play an important role. Deepening our understanding of the underlying mechanisms is crucial to the identification of new therapeutic targets to allow the development of a precision medicine in a debilitating condition whose incidence is rapidly rising. Some promising results have been reached with drugs targeting the IL-4/13 pathway, eosinophils recruitment and apoptosis, but it is still unknown whether these drugs also have the ability to prevent the remodeling processes or even revert it.

Finally, the identification of the mechanisms involved in the recognition of external antigens and the subsequent development of diagnostic tests directed to the identification of external triggers is a pivotal, yet still unanswered, goal in the management of these patients.

## Author Contributions

FR proposed the review and the layout, contributed to all sections and was in charge of the assembly of the individual paragraphs and final corrections. EH, GWC, and AR were in charge of the project and the layout, directed the aims and bibliography, distributed roles and supervised and corrected the final work. GaP, GC, EV, EC, GiP, MM, EN, and CP contributed to individual paragraphs and associated bibliography and revised the final work. All authors agreed to be accountable for the content of the work.

## Conflict of Interest

The authors declare that the research was conducted in the absence of any commercial or financial relationships that could be construed as a potential conflict of interest. The handling editor declared a past collaboration with several of the authors GWC and EH.

## Publisher’s Note

All claims expressed in this article are solely those of the authors and do not necessarily represent those of their affiliated organizations, or those of the publisher, the editors and the reviewers. Any product that may be evaluated in this article, or claim that may be made by its manufacturer, is not guaranteed or endorsed by the publisher.

## Footnotes

1 Clinicaltrials.gov Available online at: https://clinicaltrials.gov/ct2/show/NCT02353078

2 Clinicaltrials.gov Available online at: https://clinicaltrials.gov/ct2/show/NCT03473977

3 Clinicaltrials.gov Available online at: https://clinicaltrials.gov/ct2/show/NCT04543409

4 FDA Food and Drug Administration. Available online at: https://www.accessdata.fda.gov/scripts/opdlisting/oopd/detailedIndex.cfm?cfgridkey=670018

5 Clinicaltrials.gov Available online at: https://clinicaltrials.gov/ct2/show/NCT04835168

6 FDA U. S. Food and Drug Administration. Available online at: https://www.accessdata.fda.gov/scripts/opdlisting/oopd/detailedIndex.cfm?cfgridkey=408513

7 European Medicines Agency [EMA]. Available online at: https://www.ema.europa.eu/en/medicines/human/orphan-designations/eu3131205

8 FDA Food and Drug Administration. Available online at: https://www.accessdata.fda.gov/scripts/opdlisting/oopd/detailedIndex.cfm?cfgridkey=443314

9 Clinicaltrials.gov Available online at: https://clinicaltrials.gov/ct2/show/NCT04753697

10 FDA U.S. Food and Drug Administration. Available online at: https://www.accessdata.fda.gov/scripts/opdlisting/oopd/detailedIndex.cfm?cfgridkey=599317

11 Clinicaltrials.gov Available online at: https://clinicaltrials.gov/ct2/show/NCT03633617

12 Clinicaltrials.gov Available online at: https://clinicaltrials.gov/ct2/show/NCT04394351

13 Clinicaltrials.gov Available online at: https://clinicaltrials.gov/ct2/show/NCT03678545

14 Clinicaltrials.gov Available online at: https://clinicaltrials.gov/ct2/show/NCT03656380

15 FDA Food and Drug Administration. Available online at: https://www.accessdata.fda.gov/scripts/opdlisting/oopd/detailedIndex.cfm?cfgridkey=237507

16 European Medicines Agency [EMA]. Available online at: https://www.ema.europa.eu/en/medicines/human/orphan-designations/eu3161681

17 FDA Food and Drug Administration. Available online at: https://www.accessdata.fda.gov/scripts/opdlisting/oopd/detailedIndex.cfm?cfgridkey=655518

18 Clinicaltrials.gov Available online at: https://clinicaltrials.gov/ct2/show/NCT04593251

19 Clinicaltrials.gov Available online at: https://clinicaltrials.gov/ct2/show/NCT03681067

20 Clinicaltrials.gov Available online at: https://clinicaltrials.gov/ct2/show/NCT03469934

21 Clinicaltrials.gov Available online at: https://clinicaltrials.gov/ct2/show/NCT01808196

22 Clinicaltrials.gov Available online at: https://clinicaltrials.gov/ct2/show/NCT03029091

23 FDA Food and Drug Administration. Available online at: https://www.accessdata.fda.gov/scripts/opdlisting/oopd/detailedIndex.cfm?cfgridkey=839721

24 Clinicaltrials.gov Available online at: https://clinicaltrials.gov/ct2/show/NCT04248712

25 FDA Food and Drug Administration. Available online at: https://www.accessdata.fda.gov/scripts/opdlisting/oopd/detailedIndex.cfm?cfgridkey=723019

26 Clinicaltrials.gov Available online at: https://clinicaltrials.gov/ct2/show/NCT04682639

27 FDA Food and Drug Administration. Available online at: https://www.accessdata.fda.gov/scripts/opdlisting/oopd/detailedIndex.cfm?cfgridkey=668818

28 Clinicaltrials.gov Available online at: https://clinicaltrials.gov/ct2/show/NCT04322708
